# Factors related to caregivers’ risk perception of secondhand smoke exposure on children’s health

**DOI:** 10.18332/tid/143318

**Published:** 2021-12-06

**Authors:** Xavier Continente, Alejandro Rodríguez, Mónica Pérez-Ríos, Anna Schiaffino, Esteve Fernández, Maria J. López

**Affiliations:** 1 Agència de Salut Pública de Barcelona, Barcelona, España; 2 Centro de Investigación Biomédica en Red en Epidemiología y Salud Pública, Madrid, España; 3 Institut d’Investigació Biomèdica de Sant Pau, Barcelona, España; 4 Health Research Institute of Santiago de Compostela, Santiago de Compostela, Spain; 5 Department of Preventive Medicine and Public Health, University of Santiago de Compostela, Santiago de Compostela, Spain; 6 Direcció General de Planificació en Salut, Departament de Salut, Generalitat de Catalunya, Barcelona, España; 7 Tobacco Control Research Group, Bellvitge Biomedical Research Institute, Barcelona, Spain; 8 Institut Català d'Oncologia, Barcelona, España; 9 Tobacco Control Unit, Cancer Prevention and Control Program, Catalan Institute of Oncology, Barcelona, Spain; 10 Faculty of Medicine and Health Sciences, Universitat de Barcelona, Barcelona, Spain; 11 Centro de Investigación Biomédica en Red en enfermedades respiratorias, Madrid, España; 12 Departament de Ciències Experimentals i de la Salut, Universitat Pompeu Fabra, Barcelona, España

**Keywords:** child, knowledge, perception, tobacco smoke pollution

## Abstract

**INTRODUCTION:**

Home is one of the main places for children’s secondhand smoke (SHS) exposure. The implementation of smoke-free rules at home might be influenced by caregivers’ risk perception of SHS exposure. This study aimed to analyze factors related to caregivers’ risk perception of SHS exposure in children.

**METHODS:**

We conducted a cross-sectional telephone survey among a representative sample of 2411 parents or legal guardians of children aged <12 years in Spain in 2016. The main study variable was caregivers’ healthrisk perception of SHS exposure on children. We investigated correlates of risk perception using multivariate Poisson regression models with robust variance.

**RESULTS:**

Overall, 59.6% reported SHS exposure negatively affects children’s health a lot, while 34.1% that it affects quite a bit, and 5.5% and 0.8% a little bit or not at all, respectively. The factors associated with a higher caregivers’ risk perception were high education level (adjusted prevalence ratio, APR=1.11; 95% CI: 1.01–1.24), living in a non-smoking family unit (APR=1.17; 95% CI: 1.07–1.27), in a home with smoke-free rules (APR=1.34; 95% CI: 1.19–1.51), and with girls only (APR=1.14; 95% CI: 1.06–1.22).

**CONCLUSIONS:**

Caregivers’ risk perception of SHS exposure is influenced by social determinants and smoking habits in family units. These findings support the need for interventions with a social equity perspective to reduce children’s SHS exposure.

## INTRODUCTION

Children are particularly vulnerable to secondhand smoke (SHS) exposure because of their immature immune and respiratory systems and their faster breathing rates^1^. Worldwide, smoke-free policies have been progressively enacted and some countries have designed specific strategies to protect children from SHS exposure^2^.

Although households are the main place for children’s SHS exposure^3,4^, these settings are not covered in comprehensive smoke-free policies. Consequently, parental smoking at home and smokefree rules play a key role in children’s exposure to SHS^5^. According to the Health Belief Model, health-related behaviors might be influenced by risk perceptions and knowledge. Therefore, raising parental awareness of the detrimental health effects of children’s SHS exposure could decrease SHS exposure among the pediatric population^6^. At the same time, risk perception can be influenced by sociodemographic factors such as education level and social class. A higher educational level and those from more advantaged families have been related to a higher agreement with the adverse effects of SHS exposure in children^7^. However, there is scarce evidence about family determinants of parental risk perception of SHS exposure in children. This study aimed to analyze factors related to caregivers’ healthrisk perception of SHS exposure in children.

## METHODS

A cross-sectional study was performed among a country-wide representative sample of 2411 households with children aged <12 years in Spain in 2016. Detailed methods are described elsewhere^8^. Briefly, we estimated a theoretical sample of 2411 families. To achieve a representative sample of the Spanish children population younger than 12 years, we used a quota sampling method. We defined quotas by sex (girls, boys) and age (0–1, 2–3, 4–5, 6–7, 8–9, 10–11 years) of the youngest child at home; size of municipality (<10000; 10001–20000; 20001–50000; 50001–100000; 100001–200000; 200001–500000; 500001–1000000; >1000000 inhabitants) and region of Spain (the 17 Autonomous Communities). Participants were contacted using randomly selected telephone numbers according to the pre-established quotas. Those having at least one child aged <12 years and agreeing to participate were enrolled in the study.

We conducted computer-assisted telephone interviews with one adult (mother, father, or legal guardian) in each home through landlines (obtained from national directories) and mobile phones (valid phone numbers obtained at random). A questionnaire was administered by means of a structured interview that lasted on average 12 minutes.

Caregivers were asked about their perception of the health risk of SHS exposure in children through the following 4-point Likert scale question: ‘How much do you think exposure to SHS negatively affects children’s health?’. Subsequently, this variable was recoded into two categories: ‘not at all/a little bit/ quite a bit’ versus ‘a lot’.

Independent variables were the presence of at least one smoker in the home (yes/no), non-smoking rules inside the home (yes/no), and sociodemographic variables. Household sociodemographic variables consisted of the highest educational level of the main earner (primary or less, secondary, or university), the sex of all children at home (only boys, only girls, or boys and girls) and the number of children aged <12 years in the household. Additionally, some variables related to the respondent (sex, age, and country of birth) and the youngest child of the household (age and presence of asthma) were also included.

### Statistical analysis

Percentages and their 95% confidence intervals (95% CI) of high health-risk perception of SHS exposure in children were calculated, stratifying by all independent variables studied. As proposed by some authors^9^, we conducted bivariate and multivariate Poisson regression models with robust variance to compute the adjusted prevalence ratios (APR) and 95% CI of higher health-risk perception of SHS exposure. The multivariate model was adjusted for all independent variables included in the study. Missing values (<0.5% for all variables) were excluded from analyses. All statistical analyses were performed using Stata v13.1.

## RESULTS

Among the 2411 caregivers interviewed, 61.8% were women and the median age was 41 years (IQR: 37–46). About 14.8% had primary schooling or less, 40.8% had secondary school education, and 44% university education. There was at least one smoker in 29.1% of the households, and 84.5% were homes with smoke-free rules (data not shown).

Overall, 59.6% of interviewees believed that SHS exposure negatively affects children’s health a lot, 34.1% quite a bit, and 5.5% and 0.8% a little bit or does not affect children’s health at all, respectively. On multivariate analysis, higher educational level of the main earner (university education: APR=1.11; 95% CI: 1.01–1.24), living in a non-smoking family unit (APR=1.17; 95% CI: 1.07–1.27) and living in a home with smoke-free rules (APR=1.34; 95% CI: 1.19–1.51) were associated with caregivers’ high-risk perception of SHS exposure on children’s health. Caregivers living in households with only girls (APR=1.14; 95% CI: 1.06–1.22) more frequently reported a higher health-risk perception of SHS exposure than those living in households with only boys (Table 1).

**Table 1:** Prevalence of caregivers’ high awareness of the risk of SHS exposure on children’s health and family characteristics related to caregivers’ high-risk perception (a lot; reference group: not at all/a little bit/quite a bit), Spain, 2016

*Characteristics*	*n*	*Caregivers’ high-risk perception of SHS exposure on children’s health^a^*		
		*% (95% CI)*	*PR (95% CI)*	*APR^b^ (95% CI)*
**Education level of the main earner**				
Primary or less	357	55.2 (50.0–60.3)	1	
Secondary	985	57.6 (54.4–60.6)	1.04 (0.94–1.16)	1.03 (0.93–1.15)
University	1060	63.4 (60.4–66.2)	1.15 (1.04–1.27)	1.11 (1.01–1.24)
**Presence of smokers at home**				
No	1709	63.4 (61.1–65.6)	1.25 (1.15–1.36)	1.17 (1.07–1.27)
Yes	702	50.6 (46.9–54.3)	1	
**Smoke-free rules at home**				
No	375	43.7 (38.8–48.8)	1	
Yes	2036	62.6 (60.4–64.6)	1.43 (1.27–1.61)	1.34 (1.19–1.51)
**Sex of all children at home**				
Only boys	989	55.7 (52.6–58.8)	1	
Only girls	938	63.3 (60.2–66.4)	1.14 (1.06–1.22)	1.14 (1.06–1.22)
Boys and girls	484	60.5 (56.1–64.8)	1.09 (0.99–1.19)	1.08 (0.96–1.20)
**Age of the youngest child** (years)				
0–3	736	62.2 (58.7–65.7)	1.05 (0.97–1.14)	
4–7	804	58.0 (54.5–61.3)	0.98 (0.91–1.06)	
8–11	871	59.0 (55.7–62.2)	1	

CI: confidence interval. PR: crude prevalence ratio. APR: adjusted prevalence ratio. a Having reported that exposure to SHS negatively affects children’s health a lot. b Model adjusted for age, sex, and country of birth of the person interviewed, the presence of asthma in the youngest child and the number of children aged <12 years in the household.

## DISCUSSION

Our results show that 4 in 10 caregivers do not highly perceive the harmful effects of children’s SHS exposure. The factors associated with higher risk perception of SHS exposure in children were higher educational level, living in a non-smoking family unit, in a home with smoke-free rules, and with only girls.

The efforts made to implement new tobacco control policies in Europe in the last few decades have positively impacted the population’s awareness of the risk of SHS exposure to health outcomes^10^. However, social determinants might be related to the degree of awareness, with some groups being less likely to be familiar with the harmful effects of children’s exposure to SHS. Our results show that people with high educational level had a higher health-risk perception of SHS exposure. In the same line, literature shows that children from families from a lower socioeconomic position are more likely to be exposed to SHS, which, at the same time, has been linked to lower health-risk perceptions and less negative attitudes towards SHS^11^. Parents who underestimate the harmful health effects of SHS on children are more likely to smoke in their presence^12^. This concurs with the association found in this study between caregivers’ perception and the implementation of smoke-free homes independently of smoking status. Moreover, a study carried out among disadvantaged caregivers found that despite their awareness of the risk of smoking, they did not acknowledge that SHS exposure was linked to poor health outcomes in children^13^.

Our results show that caregivers from non-smoking family units (households without smoker residents) were more aware of the harmful effects of SHS than caregivers from smoking family units. The presence of smokers at home might have a greater influence on beliefs and perceptions towards smoking behaviors even among non-smokers, who might have normalized tobacco consumption in their family context and thus might underestimate the risk of SHS exposure.

We also found gender differences in risk perception of SHS exposure. Caregivers from households with only girls reported higher health-risk perceptions than those living with only boys. Caregivers’ behaviors, regardless of their biological sex, differed depending on their children’s biological sex. A hypothesis could be that caregivers are more protective of girls and feel greater responsibility for their safety^14^. In our study, when answering questions about risk perception of children’s exposure to SHS, caregivers might take their own children as a reference, with those who have girls being more sensitive to the belief that exposure to SHS in children is a strong health hazard.

### Strengths and limitations

The main strength of the study is that it analyses the potential association between social determinants and caregivers' health-risk perceptions of SHS exposure in a large, representative national sample of family units with children aged <12 years in a southern European country.

This study has some limitations that should be considered. Firstly, the cross-sectional nature of the study does not allow to establish causality. Secondly, risk perceptions are subjective judgements possibly influenced by survey factors such as the type of question or response options. Also, we used a single item that had not been previously validated to assess risk perception, so the validity and reliability could be threatened. Nevertheless, there is no consensus on how to measure risk perception of SHS exposure in children^15^. On the other hand, asking about risk perceptions might be sensitive to a desirability bias, especially when enquiring about children’s health. However, questionnaires are the only source available to estimate risk perceptions and we previously tested the questionnaire in a subsample to determine its comprehension by interviewees. Additionally, although we included some potential confounding variables related to the youngest child or the family unit in the analysis, we had no information available about other children in the family that might be influencing the relationships found.

## CONCLUSIONS

This study analyzed the potential association between social determinants and caregivers’ health-risk perceptions of SHS exposure in a large, representative national sample of children aged <12 years in a southern European country. Caregivers’ health-risk perception of SHS exposure on children was higher among those with higher educational level, living in a non-smoking family unit, in a home with smoke-free rules, and with girls only. These findings highlight the importance of implementing interventions with a social equity perspective that focus on the awareness of the harmful health effects of SHS exposure in children and the benefits of implanting smoke-free homes.

## Data Availability

The data supporting this research are available from the authors on reasonable request.
